# Olfaction and Pheromones: Uncanonical Sensory Influences and Bulbar Interactions

**DOI:** 10.3389/fnana.2017.00108

**Published:** 2017-11-15

**Authors:** Víctor Vargas-Barroso, Fernando Peña-Ortega, Jorge A. Larriva-Sahd

**Affiliations:** Instituto de Neurobiología, Universidad Nacional Autónoma de México, Campus Juriquilla, Querétaro, Mexico

**Keywords:** olfactory limbus, pheromone, olfaction, sensory convergence

## Abstract

The rodent main and accessory olfactory systems (AOS) are considered functionally and anatomically segregated information-processing pathways. Each system is devoted to the detection of volatile odorants and pheromones, respectively. However, a growing number of evidences supports a cooperative interaction between them. For instance, at least four non-canonical receptor families (i.e., different from olfactory and vomeronasal receptor families) have been recently discovered. These atypical receptor families are expressed in the sensory organs of the nasal cavity and furnish parallel processing-pathways that detect specific stimuli and mediate specific behaviors as well. Aside from the receptor and functional diversity of these sensory modalities, they converge into a poorly understood bulbar area at the intersection of the main- main olfactory bulb (MOB) and accessory olfactory bulb (AOB) that has been termed olfactory limbus (OL). Given the intimate association the OL with specialized glomeruli (i.e., necklace and modified glomeruli) receiving uncanonical sensory afferences and its interactions with the MOB and AOB, the possibility that OL is a site of non-olfactory and atypical vomeronasal sensory decoding is discussed.

## Introduction

**Figure 1:**
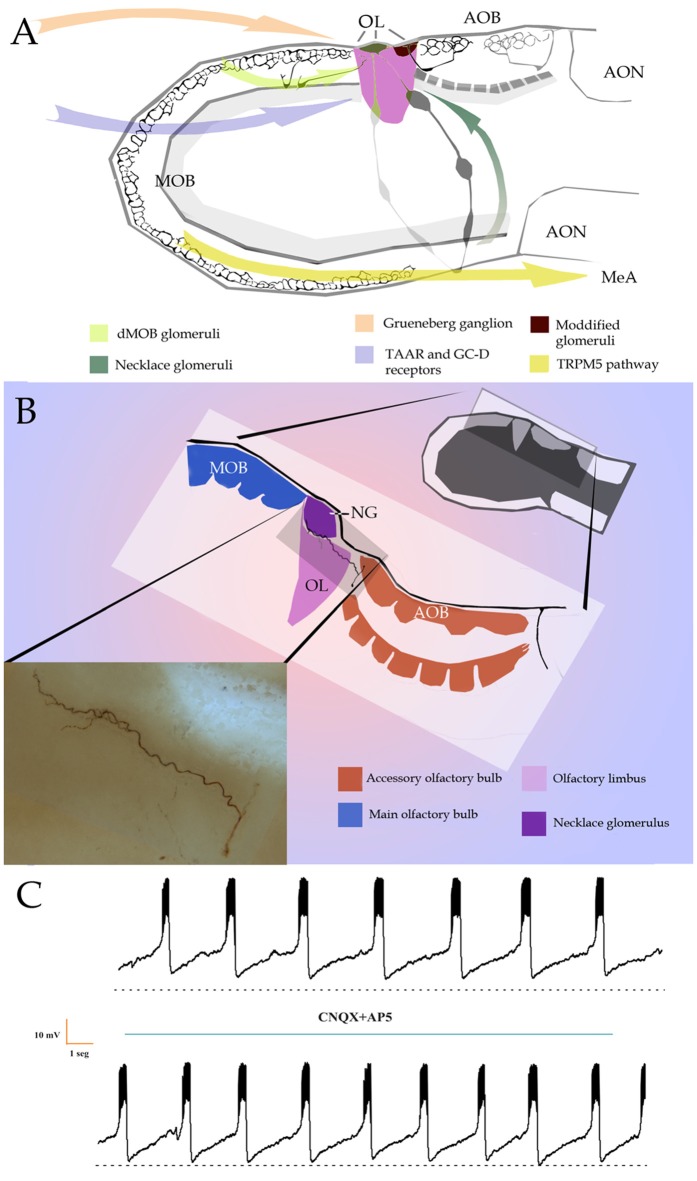
**(A)** Diagramatic representation of putative bulbar and sensory inputs to the olfactory limbus (OL) and medial nucleus of the amygdala (MeA). AON, anterior olfactory nuclei; AOB, accessory olfactory bulb; dMOB, dorsal part of the main olfactory bulb; MOB, main olfactory bulb. Arrows designate putative afferences to the OL. **(B)** Diagram of the OL (pink-colored) with the main (blue) and accessory (orange) olfactory bulbs. Insert at the bottom left. Light micrograph of a biocytin-injected large principal cell whose apical dendrites diverge to resolve, in the anterior accessory olfactory bulb (aAOB; orange) and necklace (deep purple) glomeruli. **(C)** Slice recordings of the large principal cell seen in “**B**”, insert. To note is the numerous spikes grouped into episodic bursts in a similar fashion to that observed by pacemaker neurons. Adult rat olfactory bulb.

Perception of semiochemicals in macrosmatic mammals is attributed to two sub-systems: the main (MOS) and the accessory olfactory systems (AOS), which detect volatile odors and pheromones, respectively. The MOS and AOS are regarded as anatomically and functionally independent streams of information processing; however, numerous evidence supports a combined, synergic interaction (Xu et al., 2005; Mucignat-Caretta et al., 2012; Matsuo et al., 2015). Notably, dissociated vomeronasal neurons are activated by volatile odorants (Sam et al., 2001), whereas two pheromones, 2,5-dimethyl pyrazine and 2-heptanone recruit both the olfactory epithelium (OE) and the main olfactory bulb (MOB; Lin et al., 2004). Imaging studies (Xu et al., 2005) demonstrated that mice MOB and accessory olfactory bulb (AOB) are activated by either odorants or pheromones. Further, genetically-induced loss-of-function of the dorsal part of the main olfactory bulb (dMOB) suggests that it mediates pheromone recognition (Matsuo et al., 2015). Here we overview first canonical interactions between olfactory and vomeronasal receptors with the MOB and AOB, respectively. Then, bulbar paths for non-conventional odorants and pheromones, as well as those for other sensory systems, are outlined. Lastly, the possible role of the transition between the MOB and AOB, or olfactory limbus (OL), in integrating polymodal, i.e., non-olfactory and atypical vomeronasal, sensory information is discussed (Figure 1).

### Olfactory and Vomeronasal Pathways

Sensory cells (SCs) in the OE express a single olfactory receptor (OR) from a repertoire of ~1000 OR genes (Nagayama et al., 2014). Axons from SCs expressing a given receptor project to one or two glomeruli in the MOB (Nagayama et al., 2014), which is the first information processing station for the perception of odorants (Gire et al., 2012). The glomerular neuropil gathers apical dendrites of mitral (MCs) and tufted (TCs) neurons, SCs axons, and processes of periglomerular and short-axon neurons. Receptor potentials from the OE are decoded in glomeruli to generate a coherent glomerular-output (Gire et al., 2012). SCs recruit specific sets of glomeruli in the MOB, that produce a spatial representation of olfactory stimuli (Rubin and Katz, 1999). A second processing domain within the MOB is represented by reciprocal synapses between granule cells and MCs or TCs, so that the MCs and TCs out-put is modulated by granule cells (Yokoi et al., 1995). Centrally, axons from MCs and TCs project via the lateral olfactory tract to the anterior olfactory nucleus, the olfactory tubercle, the piriform cortex, the cortical amygdala and the lateral entorhinal cortex (Sosulski et al., 2011).

Regarding the AOS, it detects mainly pheromonal cues within a cigar-shaped structure: the vomeronasal organ (VNO; Holy et al., 2000) that contains four populations of SCs distributed into two layers (Dulac and Axel, 1995). An apical layer of SCs expressing members of the vomeronasal receptor family 1 (VR1; Dulac and Axel, 1995), some members of the formyl-peptide receptor family (FPR; Rivière et al., 2009) and canonical ORs (Lévai et al., 2006); whereas basal cells express the vomeronasal receptor family 2 (V2R; Herrada and Dulac, 1997; Matsunami and Buck, 1997; Ryba and Tirindelli, 1997). Regardless of receptor expression, SCs project via vomeronasal nerves to the AOB in a segregated fashion (Schwarting and Crandall, 1991) originating anterior and posterior streams that distribute in the anterior AOB (aAOB) and posterior AOB (pAOB; Larriva-Sahd, 2008). Thus, axons from SCs in the apical VNO terminate in the aAOB, while those from the base of the VNO resolve in the pAOB (Schwarting and Crandall, 1991). Upon local information processing within the AOB, large principal cells (LPCs; Larriva-Sahd, 2008) project via the lateral olfactory tract to the medial amygdala and to the hypothalamus, modulating reproductive functions (Boehm et al., 2005) and parental behavior (Wu et al., 2014).

### Parallel Processing in the Olfactory Bulbs: Non-canonical Paths

SCs differing from “canonical” SCs by the receptor and/or signaling cascade, and by their glomerular targets, have recently been described. First, is a sub-population of SCs in the OE that utilizes guanylyl cyclase-D (GC-D) receptor and cGMP-stimulated phosphodiesterase 2 to transduce stimuli (Fülle et al., 1995). These unique SCs project to glomeruli confined to the caudal MOB-aAOB intersection: the so-called necklace glomeruli (NGs; Shinoda et al., 1989). The dMOB-aAOB interface, together with one or two NGs structure the OL (Larriva-Sahd, 2012; Figure 1). GC-D-OSCs that project to the NGs have been shown to detect the natriuretic peptides guanylin and uroguanylin, implying that this pathway may modulate water-ion homeostasis (Leinders-Zufall et al., 2007). Further, sensing of near-atmospheric levels of CO_2_ (Hu et al., 2007) and socially transmitted food preferences are also mediated by this GC-D subsystem (Munger et al., 2010). Thus, in contrast to canonical MOB glomeruli, NGs are innervated by SCs expressing distinct ORs (Mombaerts et al., 1996), although both display robust interglomerular connections (Figure 1A; Shinoda et al., 1989; Cockerham et al., 2009).

It was recently demonstrated that GC-D-OSCs located in the basal recesses of the OE express a novel family of receptors named MS4A that, unlike that of GPCRs, span SC membrane four times (Greer et al., 2016). SCs expressing this family of receptors project their axons to the region of the NGs and seem to be activated by fatty acids and a naturally aversive pheromone (Greer et al., 2016). Interestingly, Greer et al. (2016) showed that these SCs can express more than one receptor at a time. This exciting description of the MS4A putative receptors further suggests that the OL area, including the NGs, may be regarded as a “polymodal” integrative structure as it gathers inputs from assorted sensory modalities (Figures 1A,B).

Another subset of SCs from the OE has been reported to express the transient receptor potential channel M5 (TRPM5) and to be activated by putative semiochemicals (Lin et al., 2007). Additional investigations led to the characterization of a parallel circuitry involving SCs associated with gender-related social cues relevant for reproduction (Thompson et al., 2012). Namely, it was found that TRPM5-OSCs innervate ventro-medial MOB glomeruli and, in turn, MCs that receive these afferences convey the information transduced by TRPM5-OSCs to the medial amygdala (Figure 1; Thompson et al., 2012). The description of this specific MOB circuit mediating pheromonal effects is consistent with that of previous experiments defining that MCs in the ventral MOB are activated by socially relevant volatiles from conspecifics of the opposite sex (Kang et al., 2009). Moreover, MCs in the ventral MOB projected to the medial amygdala that is implicated in pheromonal responses (Kang et al., 2009, 2011). Thus, both the MOB and AOB are themselves capable of decoding a variety of specific signal molecules prior to projecting farther centrally. Altogether, these evidences strengthen the notion that the MOB and AOB, primarily committed to the detection of odorants and water-soluble pheromones, are intrinsically capable of decoding non-canonical environmental cues.

**Figure 2:**
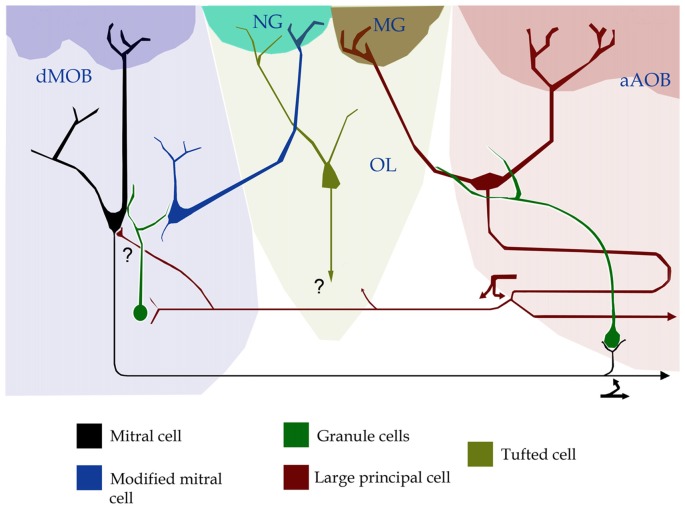
Position, interactions and putative connectivity of the olfactory limbus (OL, pale green). The OL lies between the caudal region of the main olfactory bulb (dMOB; pale blue) and the anterior part of the accessory olfactory bulb (aAOB; pink-colored). To note is that the necklace glomerulus (NG, turquois) receives dendrites from both a modified mitral cell in the dMOB and a tufted cell in the OL. A modified glomerulus (MG, dark green) receives dendrites from large principal cells (red) in the aAOB. Forked arrows designate axon collaterals of a large principal- (deep red) and a mitral-cell (black) that possibly (?) synapse with mitral- and granule-cells in the dMOB and with granule cells in the aAOB, respectively.

Still another sub-population of SCs recently described in the OE expresses trace-amine associated receptors (TAARs). TAARs are GPCRs and are thought to mediate stereotyped behaviors elicited by volatile amines (Liberles and Buck, 2006). Several sub-types of TAARs have also been identified in the Grueneberg ganglion (GG; Fleischer et al., 2007), a mass of cells in the anterior nasal cavity, assumed to be involved in chemo-sensation and thermo-sensation (Fleischer et al., 2007; Chao et al., 2014). It is interesting that, in mice, activation of SCs expressing TAAR5 mediate con-specific attraction through the detection of trimethylamine (Lin et al., 2013) and TAAR4 triggers avoidance which mediated by the detection of a carnivore odorant (2-phenylethylamine; Ferrero et al., 2011). Additionally, axons of TAAR-expressing SCs resolve in dMOB glomeruli (Johnson et al., 2012), a territory that receives axon collaterals from the aAOB (Figure 1; Vargas-Barroso et al., 2016). Lastly, all SCs expressing TAARs project to dorsomedial MOB glomeruli (Johnson et al., 2012; Pacifico et al., 2012); whereas all GG neurons, which also express V2r83 receptors of the V2R family (Fleischer et al., 2006), project to the NG territory (Storan and Key, 2006). In brief, axons of SCs detecting specific sorts of stimuli, thought to be implicated in a variety of innate behaviors converge in the atypical glomeruli that crown the OL (Figures 1A, 2).

Overall, the discovery of gene families encoding receptors that bind specific non-canonical stimuli adds up to the already complex organization of the MOS and AOS (Fülle et al., 1995; Liberles and Buck, 2006; Leinders-Zufall et al., 2007; Johnson et al., 2012; Larriva-Sahd, 2012; Thompson et al., 2012; Greer et al., 2016). Incidentally, the view that flight or fight behaviors are mediated by the MOB was unsuspected some decades ago (Raisman, 1971; Baxi et al., 2006). Hence, the notion of a clear-cut system segregation of odor and pheromone-detecting functions (Raisman, 1971; Baxi et al., 2006), should be re-evaluated. Overlapping responses and converging pathways within the olfactory bulbs (Vargas-Barroso et al., 2016) offer alternative substrates to understand influences of uncanonical sensory cues (see above) upon the MOS and AOS (Larriva-Sahd, 2012; Nicol et al., 2014; Matsuo et al., 2015; Greer et al., 2016), as commented next.

## Olfactory Limbus: A Cross Road?

While integrated responses motivated by odorant and/or pheromonal stimuli are customarily attributed to axonal convergence beyond the olfactory bulb (see Mohedano-Moriano et al., 2012; Keshavarzi et al., 2015), recent evidences support the existence of an intra-bulbar network (Figure 1; Pardo-Bellver et al., 2017). A direct link between the dMOB and AOB was first suspected by experimental lesioning of the former, leading to orthograde degeneration in the latter (Larriva-Sahd, 2008); then, reciprocal interactions between them have been characterized (Vargas-Barroso et al., 2016; see Martinez-Garcia et al., 1991). Indeed, a set of LPCs is antidromically activated by stimulation of the dMOB (Vargas-Barroso et al., 2016). Moreover, dendrites from LPCs structure both AOB and OL glomeruli, suggesting that information transduced by distinct receptor families, or even by different sensory organs, converge into LPCs (Vargas-Barroso et al., 2016, see Figure 4i therein). Additionally, LPCs projecting to the dMOB, exhibit electrophysiological characteristics of pace-maker neurons (Vargas-Barroso et al., 2016), suggesting that pheromones recruiting specific AOB glomeruli might sharpen the dMOB activity (Figure 2; see Matsuo et al., 2015).

As discussed earlier, the OL receives afferent information from a variety of sensory organs and neurons expressing all known OR families (see above; Fülle et al., 1995; Liberles and Buck, 2006; Leinders-Zufall et al., 2007; Johnson et al., 2012; Larriva-Sahd, 2012; Thompson et al., 2012; Greer et al., 2016). First, neurons expressing TAARs have been reported in the OE and the GG and shown to project to the postero-dorsal region of the MOB that includes the NGs (Storan and Key, 2006; Johnson et al., 2012; Pacifico et al., 2012). Second, GC-D-OSCs and MS4A-OSCs also project to the former region (Greer et al., 2016). Moreover, the aAOB receives projections from VIRs and from the ORs and FPRs known to be expressed in the VNO (see above; Lévai et al., 2006; Rivière et al., 2009). The receptor families mentioned, irrespective of their expression in cells of any of the sensory organs found in the nasal cavity and of their site of projection, have all been found to mediate social signals far more complex than odorant discrimination, such as thermo-sensation (Chao et al., 2014), identification of specific nutrients and/or aversive cues (Greer et al., 2016), infectious diseases (Rivière et al., 2009) and aggression (Johnson et al., 2012).

In brief, the strategical position of the OL between the dMOB and the aAOB, where afferences of SCs (i.e., TAAR, GC-D, MS4A, FPRs) constituting parallel processing-pathways distinctly innervate NGs or modified glomeruli (MGs, Greer et al., 1982) together with the demonstration that extensive interglomerular connections exist therein (Cockerham et al., 2009), suggests that the OL is involved in the processing of specific stimuli that signal relevant environmental cues (Hu et al., 2007; Leinders-Zufall et al., 2007). The latter may imply that non-olfactory and atypical vomeronasal sensory decoding may occur in the OL and, furthermore, that it is involved in the plasticity and learning of socially transmitted information, as has been shown recently (Nicol et al., 2014). Although the output(s) of the OL deserves to be investigated, it is known that dendrites organizing MGs belong to LPCs in the aAOB, which, in turn, project to the dMOB (Vargas-Barroso et al., 2016). The fundamental issue of whether principal cells of the OL project centrally or axons remain in the olfactory bulb, deserves further research.

## Author Contributions

All authors contributed equally in writing the final version of the manuscript. JL designed diagrams shown in Figures 1, 2.

## Conflict of Interest Statement

The authors declare that the research was conducted in the absence of any commercial or financial relationships that could be construed as a potential conflict of interest.
